# Co-Delivery of Gemcitabine and Honokiol by Lipid Bilayer-Coated Mesoporous Silica Nanoparticles Enhances Pancreatic Cancer Therapy via Targeting Depletion of Tumor Stroma

**DOI:** 10.3390/molecules29030675

**Published:** 2024-01-31

**Authors:** Dan Liu, Linjiang Wang, Henan Li, Dong Li, Jianwen Zhou, Jing Wang, Qi Zhang, Defu Cai

**Affiliations:** Institute of Medicine and Drug Research, Qiqihar Medical University, Qiqihar 161006, China; liudan@qmu.edu.cn (D.L.); lingjiangwang6@gmail.com (L.W.); henanli0902@gmail.com (H.L.); lidong201809@gmail.com (D.L.); zhoujianwen@qmu.edu.cn (J.Z.); wangjing@qmu.edu.cn (J.W.)

**Keywords:** gemcitabine, honokiol, lipid bilayer-coated mesoporous silica nanoparticles, syndecan-1, targeting tumor stroma

## Abstract

Syndecan-1 (SDC1) modified lipid bilayer (LB)-coated mesoporous silica nanoparticles (MSN) to co-deliver gemcitabine (GEM) and honokiol (HNK) were prepared for the targeting treatment of pancreatic cancer. The encapsulation efficiencies of GEM and HNK in SDC1-LB-MSN-GEM/HNK were determined to be 60.3 ± 3.2% and 73.0 ± 1.1%. The targeting efficiency of SDC1-LB-MSN-GEM/HNK was investigated in BxPC-3 cells in vitro. The fluorescence intensity in the cells treated with SDC1-LB-MSN-Cou6 was 2-fold of LB-MSN-Cou6-treated cells, which was caused by SDC1/IGF1R-mediated endocytosis. As anticipated, its cytotoxicity was significantly increased. Furthermore, the mechanism was verified that SDC1-LB-MSN-HNK induced tumor cell apoptosis through the mitochondrial apoptosis pathway. Finally, the biodistribution, tumor growth inhibition, and preliminary safety studies were performed on BALB/c nude mice bearing BxPC-3 tumor models. The tumor growth inhibition index of SDC1-LB-MSN-GEM/HNK was 56.19%, which was 1.45-fold and 1.33-fold higher than that of the free GEM/HNK and LB-MSN-GEM/HNK treatment groups, respectively. As a result, SDC1-LB-MSN-GEM/HNK combined advantages of both GEM and HNK and simultaneously targeted and eliminated pancreatic cancerous and cancer-associated stromal cells. In summary, the present study demonstrated a new strategy of synergistic GEM and HNK to enhance the therapeutic effect of pancreatic cancer via the targeting depletion of tumor stroma.

## 1. Introduction

Gemcitabine (GEM), a nucleoside analogue, is a first-line chemotherapy drug for pancreatic cancer. However, the median survival was 6.8 months and the objective response rate was 9.4% for GEM-treated patients [1]. The reason for the worse prognosis of pancreatic cancer in clinic is the resistance to GEM [2]. This resistance resulted mainly from the stroma of pancreatic cancer in which tumor cell clusters are wrapped by a dense, interconnected stromal structure, creating physical barriers to prevent GEM from penetrating and accumulating in the tumor cells [3,4]. Insufficient activation or rapid inactivation may lead to unfavorable GEM pharmacokinetics [5]. In recent years, stromal directed agents that break the dense stromal microenvironment have been introduced into the clinic to improve GEM delivery [6]. The co-administration showed an improvement in the survival of metastatic pancreatic cancer patients, but GEM was more toxic and restricted to a few patients with good performance status. Therefore, there is an urgent need for a new strategy to reduce the toxicity of GEM, improve drug delivery efficiency to the tumor site, and subsequently increase antitumor capacity while using combination chemotherapy. 

Honokiol (HNK) is a natural phenolic compound extracted from the root, bark, and leaves of *Magnolia grandiflora* [7,8]. HNK can inhibit the growth and proliferation of many tumor cell lines, such as breast, lung, gastric cancer, multiple myeloma, prostate cancer, liver, pancreatic cancer, skin cancer, and so on. The anticancer mechanism studies revealed that HNK acted on multiple molecular targets that regulate the expression of proteins controlling the relative pathway of cancer. To be specific, HNK can decrease expression of Bcl-2, MMP, P-glycoprotein, c-FLIP, H3K27 methyltransferase, sSRC-3, CXCR4, cyclin-B1, CDC2, CDC25C, and so on. Moreover, it can enhance the expression of Bax, Bid, Bak, BMP7, p-CDC2, p-CDC25, and more [9,10,11,12]. In addition, it has also been found to interfere with the nuclear factor kappa-light-chain-enhancer of activated B cells (NF-κB), signal transducer and activator of transcription 3 (STAT3), epidermal growth factor receptor (EGFR), mammalian target of rapamycin (mTOR), epithelial-mesenchymal transition (EMT), and Sonic hedgehog (SHH) signal pathway of the cancers [11,13,14,15]. More importantly, the combination of HNK and clinical chemotherapy drugs for cancer therapy generated better therapeutic effects [16]. Therefore, HNK may be a potential candidate compound for combined chemotherapy with GEM. However, due to the significant differences in physical and chemical properties between GEM and HNK, the traditional combination chemotherapy strategy of mixing the two independent drugs has no obvious advantages, which greatly hinders the clinical research and application of the combination for these two drugs. 

Many nanodrug delivery systems such as liposomes, micelles, and nanoparticles have been employed in multifunctional nanocarrier platforms for cancer therapy [17,18,19]. Among these, LB coating mesoporous silica nanoparticles (LB-MSN) have been widely used for delivering drugs to tumor sites due to synergistically combining the advantages of liposomes with those of mesoporous silica, including stabilized properties, improved loading capacity for multiple drugs, prolonged circulation time, enhanced tumor accumulation, and alleviated side effects of conventional chemotherapy [20,21]. In addition to combining the independent advantages of the MSN and the LB systems, LB-MSN could suppress large-scale membrane bilayer fluctuations responsible for LB instability and leakage, while the LB also served to retain soluble cargos within the MSN [22]. More importantly, the phospholipid bilayer coated on the MSN allowed further functionalization with the targeting ligands to improve dispersibility of LB-MSN in biological media and facilitate its accumulation in tumor cells [23].

It is generally recognized that the tumor microenvironment plays a crucial role in tumorigenesis and treatment success. The tumor stroma is one of the main barriers for drug delivery, especially for nanodrug delivery systems. Targeting the tumor stroma, in combination with chemotherapy, acting both on tumor cells and the tumor microenvironment is a promising option for the treatment of pancreatic cancer [24,25]. Insulin-like growth factor-1 receptor (IGF1R) is a tyrosine kinase receptor that is usually highly expressed in pancreatic tumors and stromal cells and lowly expressed in normal pancreatic tissues [3]. This feature makes IGF1R a potential target for therapeutic strategy targeting pancreatic cancer and its stroma. Syndecan-1 (SDC1), a type of heparan sulfate proteoglycan composed of a 310 amino acids long core protein, has been shown to specifically bind to IGF1R [26,27]. Thus, SDC1 is an appropriate ligand for developing a targeting LB-MSN nanoparticle drug delivery system for improving targeted therapy of stromal abundant pancreatic cancer through SDC1/IGF1R receptor-mediated endocytosis. 

**Scheme 1:**
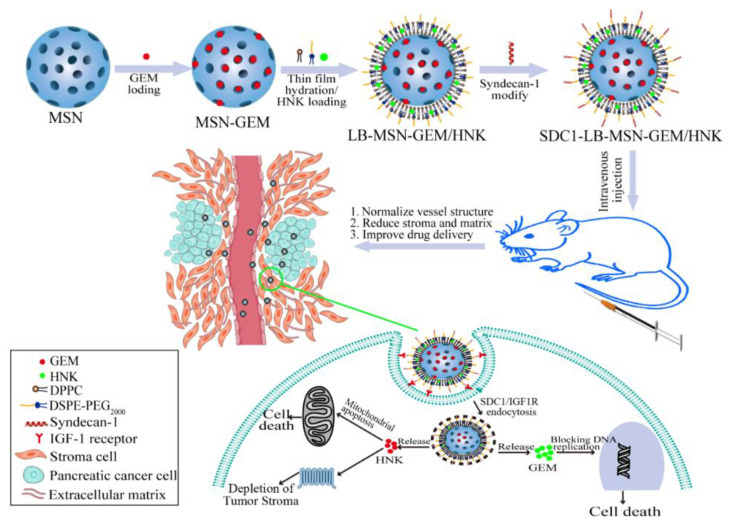
Schematic diagram of SDC1-LB-MSN-GEM/HNK for targeted delivery of GEM and HNK in the treatment of pancreatic cancer.

In this study, we innovatively fabricated LB-coated MSN nanoparticles to co-deliver GEM and HNK (LB-MSN-GEM/HNK) for the targeting treatment of pancreatic cancer. The hydrotropic GEM was encapsulated into the pores of the MSN, and the pores were sealed by LB entrap to ensure a high GEM load. At the same time, the hydrophobic drug HNK was incorporated into the LB, which can deplete the dense stromal microenvironment and synergistically with GEM on pancreatic tumor cells to inhibit tumor growth. SDC1 was employed to further improve the stroma and tumor targeting of LB-MSN-GEM/HNK, in which the uptake of GEM and HNK were significantly increased through SDC1/IGF1R receptor-mediated endocytosis. SDC1-LB-MSN-GEM/HNK combined advantages of both GEM and HNK simultaneously targeted and eliminated pancreatic cancerous and cancer-associated stromal cells under the help of nanoparticles carrier may provide a new strategy for efficacious treatment of pancreatic cancer (Scheme 1). 

## 2. Results and Discussion

### 2.1. Preparation and Characterization of SDC1-LB-MSN-GEM/HNK

**Figure 1:**
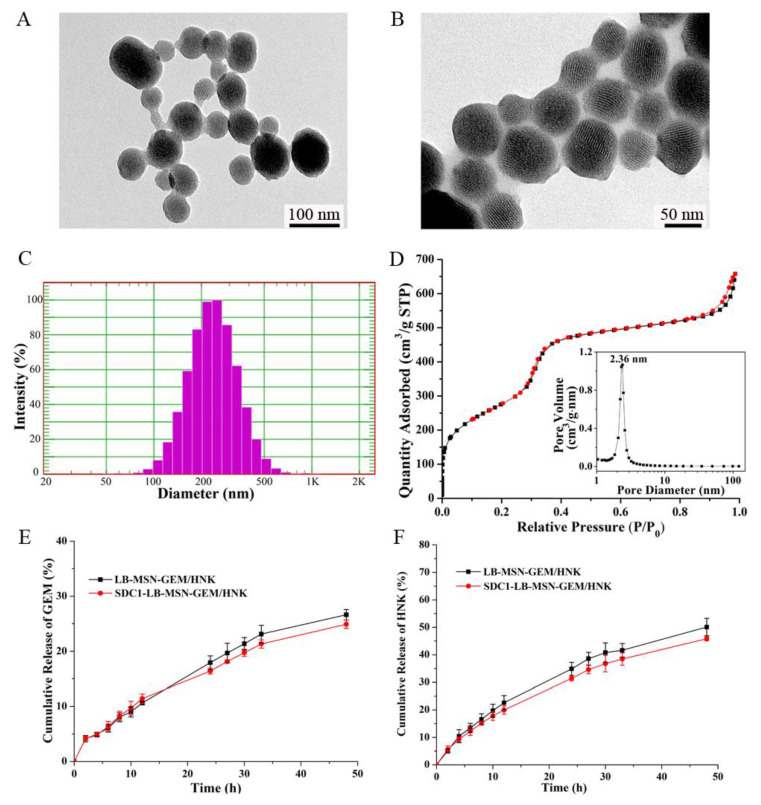
TEM images of MSN (**A**) and LB-MSN-GEM/HNK (**B**). Size distribution graph of LB-MSN-GEM/HNK (**C**). N_2_ adsorption-desorption isotherms and pore size distributions of MSN (**D**). The release curves of GEM (**E**) and HNK (**F**) from LB-MSN-GEM/HNK and SDC1-LB-MSN-GEM/HNK.

The synthetic process of the SDC1-LB-MSN-GEM/HNK is summarized in Scheme 1. First, MSN nanoparticles were synthesized and encapsulated GEM into MSN (MSN-GEM). Second, the lipid bilayer with HNK was coated on the surface of MSN-GEM by a thin film dispersion hydration method to obtain the LB-MSN-GEM/HNK. Finally, syndecan-1 was grafted to the LB-MSN-GEM/HNK nanoparticles by an amidation reaction. The MSN exhibited an array of well-ordered mesoporous and spherical morphology (Figure 1A), and the diameters of the MSN were 70–80 nm. The texture parameters of the MSN were measured by the low-temperature nitrogen adsorption-desorption analysis (Figure 1D). The results indicated that the MSN exhibited a high specific surface area with 1012.2 m^2^/g, a mesoporous diameter of 2.36 nm, and total pore volume is 0.87 cm^3^/g. The high specific surface area and total pore volume make MSN suitable for drug carriers. The TEM image of LB-MSN-GEM/HNK demonstrated the uniform coating of the nanoparticle surface by the lipid bilayer, and the encapsulation of GEM and HNK did not affect the MSN structure (Figure 1B). It can be seen that the particle size distribution of LB-MSN-GEM/HNK was narrow with a diameter of 235.2 nm from the DLS result in Figure 1C, which was about 3 times of MSN. The reason might be that the LB coated onto two or three MSN particles and formed an LB-MSN-GEM/HNK unit. The LB-MSN-GEM/HNK displayed a negative zeta potential of −48.74 mV, while the zeta potential increased to −21.02 mV after SDC1 conjugation due to the positive charge of SDC1, implying that the SDC1 was successfully conjugated on the LB-MSN-GEM/HNK. Moreover, none of free SDC1 protein band was detected in the SDS-PAGE with Coomassie blue staining assay, further confirming that SDC1 was conjugated on the LB-MSN-GEM/HNK by a chemical bond. The encapsulation efficiencies of GEM and HNK in nanoparticles were determined to be 60.3 ± 3.2% and 73.0 ± 1.1%, respectively, by using UPLC.

The release profiles of drugs from LB-MSN-GEM/HNK and SDC1-LB-MSN- GEM/HNK are shown in Figure 1E,F. It was clear that the releases of GEM and HNK from nanoparticles were time-dependent. For GEM release, no initial burst release pattern was observed in either LB-MSN-GEM/HNK or SDC1-LB-MSN-GEM/HNK, which revealed that GEM was encapsulated mainly in the mesoporous of the MSNs, and LB served as a protective barrier to prevent the GEM release. The cumulative release rates of GEM were 26.6% for LB-MSN-GEM/HNK and 24.9% for SDC1-LB-MSN-GEM/HNK at 48 h, exhibiting a remarkably prolonged release. The release of HNK exhibited a similar sustained release feature in both LB-MSN-GEM/HNK and SDC1-LB-MSN-GEM/HNK. The release rates of HNK were 50.1% and 45.8% for LB-MSN-GEM/HNK and SDC1-LB-MSN-GEM/HNK at 48 h, respectively. Taken together, the SDC1-LB-MSN-GEM/HNK can effectively reduce the drug release in blood circulation, reducing the side effects of GEM. The prolonged release behavior of SDC1-LB-MSN-GEM/HNK is favorable for achieving efficient GEM and HNK co-delivery and generating strong chemotherapeutic combination therapy on pancreatic tumors. Meanwhile, no significant difference was observed for the releases of GEM and HNK from the two groups, indicating that SDC1 modification did not affect the drug release from the nanoparticles. 

### 2.2. In Vitro Cellular Uptake of Cou6-Labeled Nanoparticles

**Figure 2:**
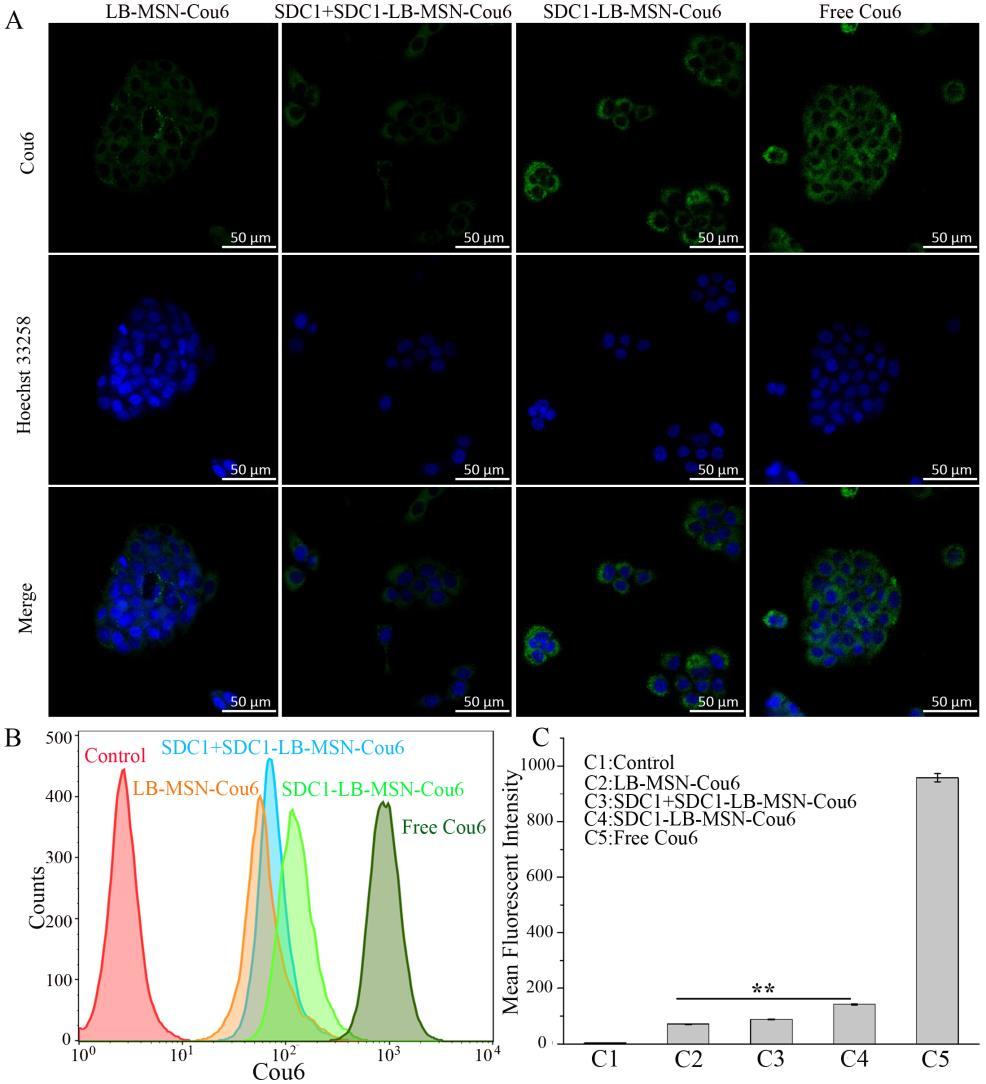
(**A**) Confocal laser scanning microscopy images of BxPC-3 cells. (**B**) Flow cytometric quantitative determination of Cou6 uptake by BxPC-3 cells. (**C**) Mean fluorescence intensity of Cou6 in BxPC-3 cells measured by flow cytometry. Each bar represents mean fluorescence intensity ± SD (*n* = 3), ** *p <* 0.01.

The cellular uptake is a prerequisite for drugs delivered by nanoparticles to take pharmacological effect. Therefore, both qualitative and quantitative cellular uptake analyses were performed to evaluate the cancer cell targeting efficacy of SDC1-LB-MSN nanoparticles. As demonstrated in Figure 2A, confocal images of cellular uptake of Cou6-loaded nanoparticles showed that both LB-MSN-Cou6 and SDC1-LB-MSN-Cou6 can enter BxPC-3 cells. As a positive control, the Cou6 group exhibited the strongest green fluorescence intensity in all groups. Compared with the LB-MSN-Cou6 group, the BxPC-3 cells treated with SDC1-LB-MSN-Cou6 showed significantly higher green fluorescence intensity, implying an efficient tumor cell targeting drug delivery for the SDC1-LB-MSN-Cou6. Competition experiments were carried out to verify whether SDC1-LB-MSN-Cou6 enters BxPC-3 cells through SDC1/IGF1R receptor-mediated endocytosis. Excessive free SDC1 was added to block the IGF1R receptor highly expressed on the surface of the BxPC-3 cells before incubation of the cells with SDC1-LB-MSN-Cou6. In the SDC1+SDC1-LB-MSN-Cou6 group, the BxPC-3 cells showed a similar fluorescent intensity to the LB-MSN-Cou6 treated ones, implying the excessive free SDC1 blocked the IGF1R receptor and decreased the cellular internalization efficiency of SDC1 modified nanoparticles, which further verified that SDC1-LB-MSN-Cou6 entered cancer cells through SDC1/IGF1R receptor-mediated endocytosis. Moreover, the quantitative analysis of the cellular uptake was measured via flow cytometry (Figure 2B,C); SDC1-LB-MSN-Cou6 showed higher cellular uptake into the BxPC-3 cells than non-targeting ligand-conjugated nanoparticles LB-MSN-Cou6 and the competition group. Specifically, the mean Cou6 fluorescent intensities of the LB-MSN-Cou6 and SDC1-LB-MSN-Cou6 group were 70.9 and 142.7, respectively. More than 201.2% fluorescent intensity was observed in the BxPC-3 cells treated with SDC1-LB-MSN-Cou6 than the LB-MSN-Cou6-treated ones. The mean Cou6 fluorescent intensity of the SDC1+SDC1-LB-MSN-Cou6 group was 88.3, which was much lower than that of SDC1-LB-MSN-Cou6. The results were consistent with the study of cellular uptake obtained by confocal microscopy. Taking these results together, we can confirm that the cancer cell targeting characteristic of SDC1-LB-MSN is due to SDC1/IGF1R receptor-mediated endocytosis.

### 2.3. In Vitro Cytotoxicity Studies of SDC1-LB-MSN-GEM/HNK

**Figure 3:**
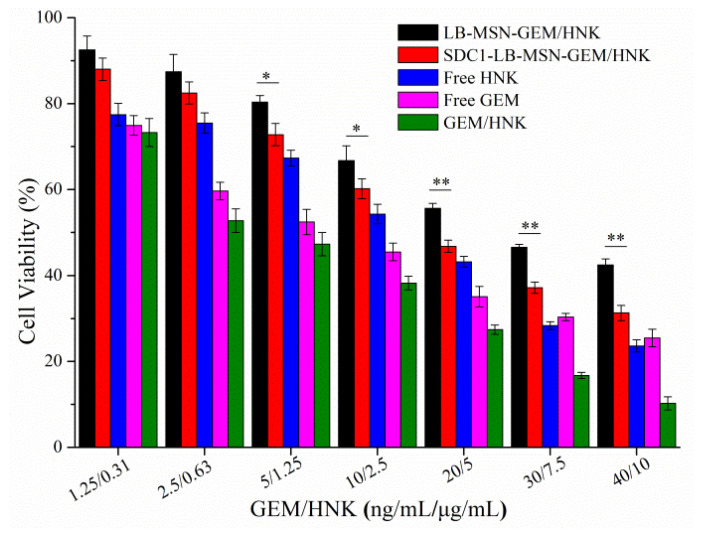
Viabilities of BxPC-3 cells after being treated by various formulations. Each bar denotes mean ± SD, (*n* = 6). * *p* < 0.05 and ** *p* < 0.01.

Cytotoxic activities of released GEM and HNK from LB-MSN-GEM/HNK and SDC1-LB-MSN-GEM/HNK toward BxPC-3 cells were evaluated by the SRB assay. As shown in Figure 3, both free drug solution and drug-loaded nanoparticles inhibited BxPC-3 cell proliferation in a dose-dependent manner. As the positive groups, the cells incubated with free GEM, HNK, and the combination of GEM with HNK (GEM/HNK) groups showed a stronger inhibitory effect among all drug formulations, which may be caused by the fact that the free drug can directly expose to BxPC-3 cells through passive diffusion. Of note, the IC_50_ of free GEM and HNK were 6.95 ng/mL and 3.37 μg/mL, while the IC50 values of GEM and HNK in the GEM/HNK group were 3.40 ng/mL and 0.82 μg/mL, respectively. The therapeutic efficacy of combination chemotherapy strategy was superior to the single free drug, indicating that the combined administration of GEM and HNK could achieve a synergistic outcome. Moreover, the IC_50_ of GEM in LB-MSN-GEM/HNK and SDC1-LB-MSN-GEM/HNK was 25.45 ng/mL and 15.99 ng/mL, and the IC_50_ of HNK in LB-MSN-GEM/HNK and SDC1-LB-MSN-GEM/HNK was 6.36 μg/mL and 4.00 μg/mL, respectively. It should be noted that cells treated with SDC1-LB-MSN-GEM/HNK showed a higher growth inhibition when compared with the ones from the LB-MSN-GEM/HNK-treated group. Such results were attributed mainly to the fact that SDC1 can specifically bind to the IGF1R receptor on the BxPC-3 cells and promote the internalization of the SDC1-LB-MSN-GEM/HNK. Thus, the SDC1-LB-MSN-GEM/HNK provides clear therapeutic potential over LB-MSN-GEM/HNK, from both efficacy and drug toxicity perspectives.

### 2.4. SDC1-LB-MSN-GEM/HNK Induces Cell Apoptosis and Relevant Mechanism

**Figure 4:**
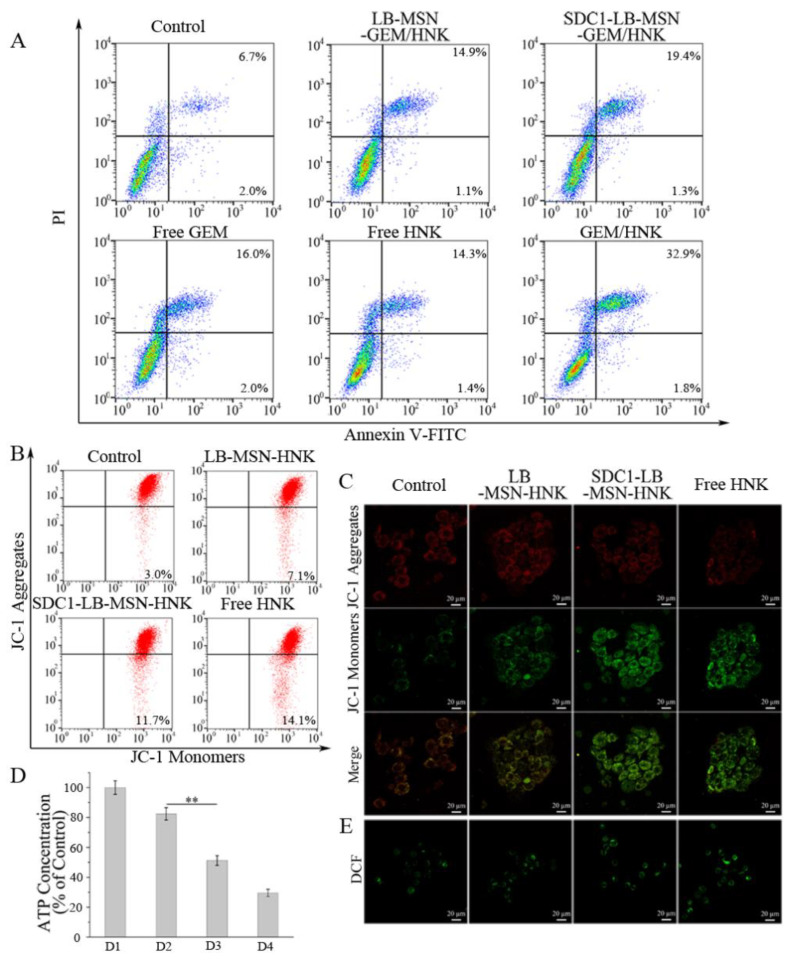
(**A**) Apoptosis results of BxPC-3 cells after being treated with various formulations for 24 h; cells were stained using Annexin V-FITC/PI and analyzed by flow cytometry. (**B**) MMP changes of BxPC-3 cells after being treated with LB-MSN-HNK, SDC1-LB-MSN-HNK, and free HNK; cells were stained with JC-1 staining and measured by flow cytometry. (**C**) MMP changes of BxPC-3 cells analyzed by laser confocal microscope; red fluorescence reflects the aggregated form of JC-1, and green fluorescence reflects the monomeric form of JC-1. (**D**) ATP levels in BxPC-3 cells treated with various formulations (D1: Control, D2: LB-MSN-HNK, D3: SDC1-LB-MSN-HNK, D4: Free HNK). (**E**) Laser confocal microscope images of ROS levels in BxPC-3 cells after being treated with various formulations for 24 h; the cells were stained with DCFH-DA, and green fluorescence reflects the substance of DCF. ** *p* < 0.01.

The apoptosis-inducing effects were quantitatively assessed using flow cytometric assay. In Figure 4A, as a positive group, the total apoptotic ratio in BxPC-3 cells treated with the combination of GEM and HNK was about 34.7%. However, the total apoptotic ratio in BxPC-3 cells after treatment with SDC1-LB-MSN-GEM/HNK was 20.7%, which was much higher than 16.0% for LB-MSN-GEM/HNK. The results demonstrated that SDC1-LB-MSN-GEM/HNK has an enhanced apoptotic induction effect, which could be attributed to the more efficient cell internalization of nanodrugs with SDC1 serving as the active targeting ligand and the activation of mitochondrial apoptotic pathway induced by the HNK.

To further elucidate the mechanism of SDC1-LB-MSN-GEM/HNK inducing cell apoptosis through the activation of the mitochondrial apoptotic pathway, we prepared SDC1-modified nanoparticles SDC1-LB-MSN-HNK without GEM and evaluated the mitochondrial function damage in BxPC-3 cells. The energy obtained from the double-membrane-structured mitochondrial respiration forces the protons in the mitochondrial matrix to pass through the inner membrane to form a mitochondrial membrane potential (MMP), which plays an important role in regulating cellular processes such as the cell apoptosis pathway, release of ROS, and synthesis of ATP [28]. Thus, we first measured the MMP by using a membrane-permeable lipophilic cationic fluorescent probe JC-1 to determine the effect of SDC1-LB-MSN-HNK on the organelle status. As shown in Figure 4B, the MMP of all experimental groups exhibited a significant decrease when compared with the control group. Especially upon treatment of SDC1-LB-MSN-HNK, the MMP of BxPC-3 cells decreased approximately 11.7%, which was almost 1.65 times higher than that of the LB-MSN-HNK group. Furthermore, such MMP change was qualitatively examined using confocal laser scanning microscopy (Figure 4C). Similarly, the BxPC-3 cells treated with HNK formulations showed obviously increased green fluorescence, implying a decline of MMP. By comparison, the decrease of MMP of SDC1-LB-MSN-HNK was more significant than that of LB-MSN-HNK.

The MMP is critical for ATP synthase to make ATP, which is the most important energy molecule that ensures the energy supplies for various life activities of cells [29]. The occurrence of a decline in MMP is typically accompanied by a decrease in ATP levels. Thus, the levels of ATP secretion release were detected after various treatments and are illustrated in Figure 4D. Compared with the PBS-treated BxPC-3 cells, the ATP levels in various HNK treated cells showed drastic decrease. Distinctly, SDC1-LB-MSN-HNK resulted in more conspicuous ATP level decrease at 51.2% benefiting from the SDC1 modification, while LB-MSN-HNK hardly resulted in ATP level decrease at only 82.4%.

The fluorescent probe DCFH-DA was used to determine the ROS in BxPC-3 cells. As identified by the confocal laser scanning microscopy results (Figure 4E), the ROS in various HNK formulation treated cells showed marked increase, evidenced by more intense and uniform green fluorescent in those BxPC-3 cells. In particular, the green fluorescence intensity of the SDC1-LB-MSN-HNK group was stronger than that in the LB-MSN-HNK group, authenticating the markedly higher ROS generation of SDC1-LB-MSN-HNK.

### 2.5. In Vivo Tumor Targeting

**Figure 5:**
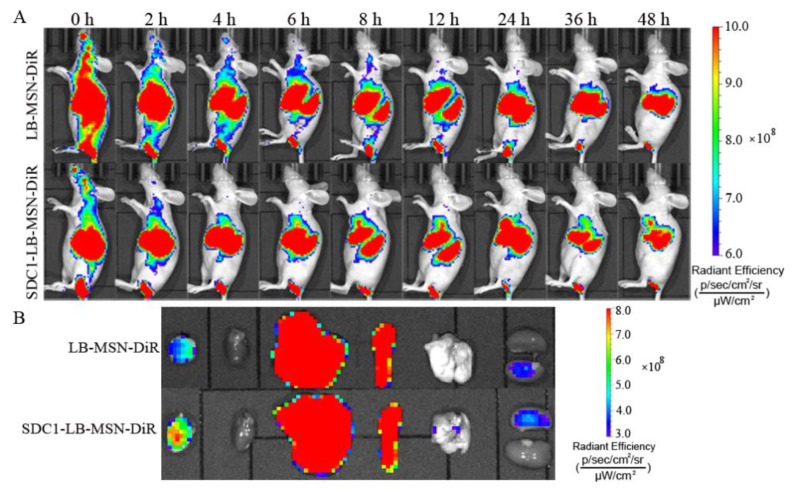
(**A**) In vivo fluorescent images of BxPC-3 tumor-bearing BALB/c nude mice at different time points after the intravenous injection with LB-MSN-DiR and SDC1-LB-MSN-DiR. (**B**) Ex vivo fluorescent images of tumors and major organs excised from BxPC-3 tumor-bearing BALB/c nude mice after intravenous injection of 48 h.

To verify the targeting efficacy of SDC1 modifying LB-MSN in pancreatic tumors, a BxPC-3 xenograft pancreatic tumor model was established. A fluorescent probe DiR labeled nanoparticles LB-MSN-DiR and SDC1-LB-MSN-DiR were injected intravenously, and the fluorescent images of mice were captured by an in vivo imaging system at a certain time interval. As shown in Figure 5A, DiR fluorescence was immediately distributed throughout the body of mice after injection of LB-MSN-DiR or SDC1-LB-MSN-DiR. As time went on, SDC1-LB-MSN-DiR had a preferential fluorescence accumulation at the tumor site than LB-MSN-DiR, owing to the SDC1 modification and the SDC1/IGF1R receptor-mediated endocytosis. Specifically, at 6 h the LB-MSN-DiR treatment mouse showed fairly strong fluorescence, and then the signal faded rapidly, presumably due to the rapid clearance of nanoparticles by the reticuloendothelial system. In contrast, the strong fluorescent signal in the mouse injected with SDC1-LB-MSN-DiR was still observable after 48 h. Subsequently, the mice were sacrificed and the major organs and tumor tissues were isolated for further ex vivo imaging (Figure 5B). As expected, the stronger fluorescence intensity was still indicated in the tumor tissue of the SDC1-LB-MSN-DiR treatment mouse at 48 h, which proved that the considerable active targeting capacity of SDC1-LB-MSN-DiR was endowed by SDC1 modification. A SDC1 modified LB-MSN drug delivery system might be a promising targeting formulation for pancreatic cancer therapy in clinic.

### 2.6. In Vivo Antitumor Efficiency

**Figure 6:**
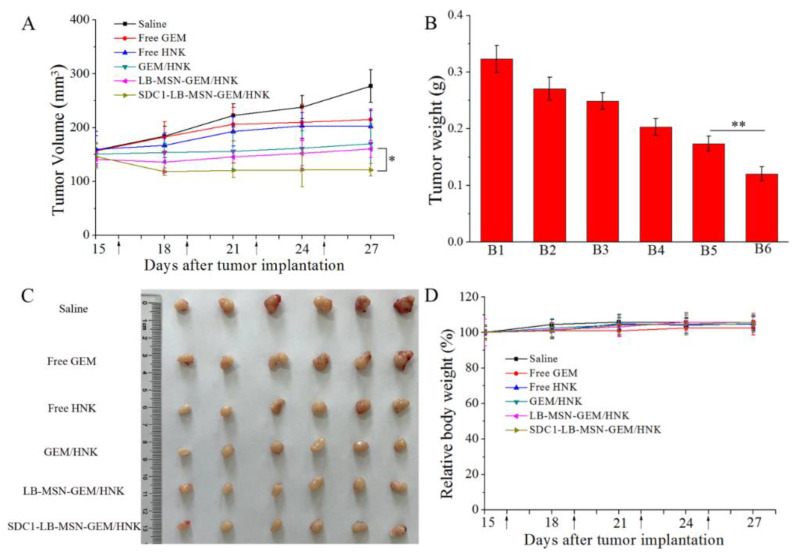
In vivo antitumor efficacy of various formulations on BxPC-3 tumor-bearing BALB/c nude mice. (**A**) Tumor growth curves. (**B**) Tumor weight change curves. B1: Saline, B2: Free GEM, B3: Free HNK, B4: GEM/HNK, B5: LB-MSN-GEM/HNK, B6: SDC1-LB-MSN-GEM/HNK. (**C**) Photographs of tumors from each treatment group excised on the 27th day. (**D**) Body weight change curves. The injection times are indicated by black arrows; each bar denotes mean ± SD, (*n* = 6), * *p <* 0.05 and ** *p* < 0.01.

BxPC-3 xenograft pancreatic tumor model was exploited to investigate the in vivo antitumor efficacy of the SDC1-LB-MSN-GEM/HNK. The tumor volumes, ex vivo tumor weights, images of ex vivo tumors, and weight changes of nude mice were recorded and analyzed during the treatment course of two weeks. As shown in Figure 6A, compared to free GEM/HNK and LB-MSN-GEM/HNK, the SDC1-LB-MSN-GEM/HNK group exhibited significant suppression on tumor growth. The tumor growth inhibition index of the mice treated with SDC1-LB-MSN-GEM/HNK was calculated to be as high as 56.19%, which was 1.45-fold and 1.33-fold higher than that of the free GEM/HNK and LB-MSN-GEM/HNK treatment groups, respectively. At the end of the experiment, the tumors were weighed (Figure 6B) and the final tumor morphology was photographed (Figure 6C). Similar trends were observed with the above results, which indicated that the antitumor effect of SDC1-LB-MSN-GEM/HNK was enhanced after SDC1 modification. In addition, the potential adverse effect of the various formulations was estimated by the fluctuation of body weight (Figure 6D). Regardless of the different treatment groups, no apparent loss in body weight of mice was found during the treatment process. The H&E staining of the major organ sections also indicated that the SDC1-LB-MSN-GEM/HNK treatment did not elicit appreciable organ damage, confirming the superior biosafety of SDC1-LB-MSN-GEM/HNK.

**Figure 7:**
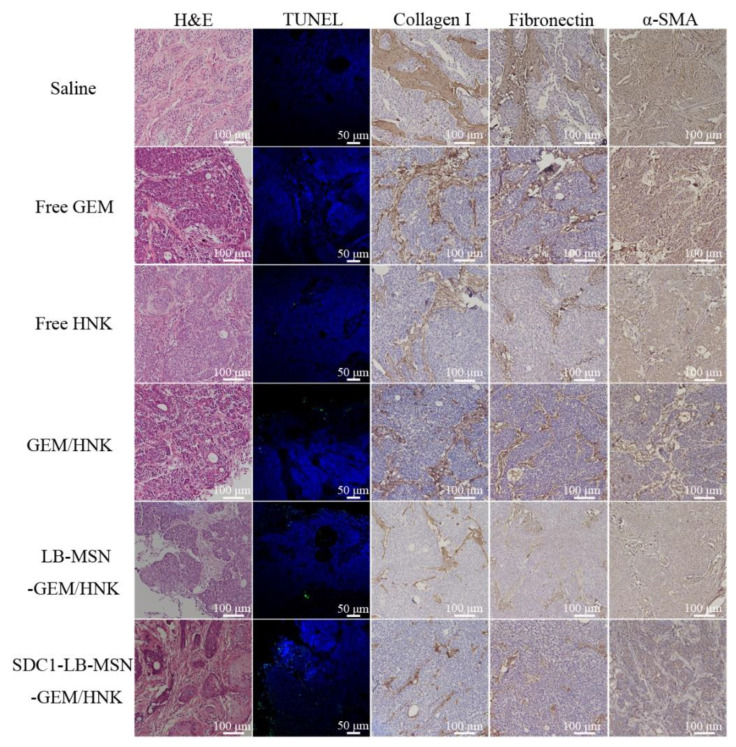
H&E and TUNEL staining of tumors, as well as IHC staining of collagen Ι, fibronectin, and α-SMA in tumors after mice were treated with various formulations for 12 days.

Collagen I is the most abundant protein in the human body; reports have identified that collagen I accumulates extensively in the microenvironment of pancreatic cancer, which consists of the extracellular matrix and cancer-associate fibroblasts [30]. A main component of the extracellular matrix is fibronectin, which is secreted by cancer-associated stromal cells, while α-smooth muscle (α-SMA) is recognized as a marker of activated cancer associated fibroblasts. Thus, the tumor slices were also immunohistochemically stained to check the collagen I, fibronectin, and α-SMA expression to verify the depletion effect of SDC1-LB-MSN-GEM/HNK on the stroma of pancreatic cancer. As found in Figure 7, the tumors treated with the saline exhibited densely aligned collagen I with a large proportion of the collagen-stained area in the tumor slice. In sharp contrast, in tumors treated with SDC1-LB-MSN-GEM/HNK, the collagen-stained area was significantly reduced, and its alignment was completely disrupted. At the same time, the expression level of fibronectin and α-SMA exhibited a similar trend also confirming the tumor stroma depletion effect of SDC1-LB-MSN-GEM/HNK on pancreatic cancer. It might be attributed to the strongest tumor-targeting delivery of SDC1-LB-MSN-GEM/HNK, followed by the release and activation of HNK as a tumor stroma regulator. 

Taken together, the in vivo results indicated that SDC1-LB-MSN-GEM/HNK possessed excellent active targeting capability, facilitating the accumulation of HNK in pancreatic cancerous and cancer-associated stromal cells. Followed by the release and activation of HNK as a tumor stroma regulator, the dense stromal microenvironment of pancreatic cancer was reversed and GEM could be delivered deep into the tumor site. Finally, GEM could interfere with the DNA synthesis of pancreatic cancer cells and HNK could induce apoptosis of pancreatic cancer cells by regulating the mitochondrial apoptosis pathway, thus leading to the optimal therapeutic effect for pancreatic cancer. 

## 3. Materials and Methods

### 3.1. Chemical Reagents

HNK (>98%) was purchased from Meilun Biotechnology (Dalian, China). GEM (>98%) was purchased from Macklin Biochemical Co., Ltd. (Shanghai, China). Tetraethylorthosilicate (TEOS) was purchased from TCI (Tokyo, Japan). Cetyltrimethylammonium bromide (CTAB), 1-(3-Dimethylaminopropyl)-3- ethylcarbodiimide hydrochloride (EDC), N-Hydroxysulfosuccinimide sodium salt (NHS), 1,2-distearoyl-snglycero-3- phosphoethanolamine-N-[methoxy(polyethylene glycol)-2000] (DSPE-PEG_2000_), and DSPE-PEG_2000_-COOH were purchased from J&K Scientific Co., Ltd. (Beijing, China). 1,2-dioleoyl-sn-glycero-3-phosphocholine (DOPC) and cholesterol (Chol) were obtained from AVT (Shanghai) Pharmaceutical Tech Co., Ltd. Coumarin-6 (Cou6), Hoechst 33258, sulforhodamine B (SRB), agarose, and 1,1’-dioctadecyl-3,3,3’,3’-tetramethylindotricar-bocyanine iodide (DiR) were obtained from Sigma-Aldrich (Shanghai, China). RPMI 1640 medium and penicillin-streptomycin were obtained from Macgene Technology Ltd. (Beijing, China). Fetal bovine serum (FBS) was obtained from Wisent Co., Ltd. (Nanjing, China). Annexin V-FITC/PI apoptosis kit, reactive oxygen species assay kit (ROS), adenosine 5′-triphosphate (ATP) assay kit, mitochondrial membrane potential assay kit with JC-1, H&E kit, and IHC assay kit were bought from Beyotime Biotechnology Ltd. (Shanghai, China). DeadEnd Colorimetric TUNEL System assay kit was bought from Promega Ltd. (Beijing, China).

### 3.2. Preparation and Characterizations of MSN and SDC1-LB-MSN-GEM/HNK

The MSN nanoparticles were synthesized according to the published literature with slight modifications [31]. In a typical synthesis, 100 mg of CTAB was dissolved in 48 mL of water under stirring, and 3.5 mL 0.2 M NaOH aqueous solution was added subsequently. The mixture was stirred for 30 min at 70 °C. Then, 0.5 mL of TEOS was added dropwise to the mixture. The solution was vigorously stirred for 4 h at 70 °C. Afterward, the product was centrifuged, washed with ethanol three times, and dried at 40 °C. Finally, the nanoparticles were calcined at 400 °C for 2 h to remove the CTAB. 

The 20 mg of MSN was mixed with 5 mL methanol solution of GEM (3 mg/mL). After evaporating the methanol, the product (MSN-GEM) was centrifuged and washed with cool water three times. The soaked MSN-GEM was resuspended in 2 mL of PBS (pH 7.4). A thin-film hydration method was used to attach a surface LB coating [5]. DOPC, cholesterol, DSPE-PEG2000, DSPE-PEG2000-COOH, and HNK (weight ratio = 20:5:4:1:2) were dissolved in the dichloromethane. The solvent was dried at 37 °C for 1 h using a rotary evaporator. The lipid film was hydrated in MSN-GEM PBS suspension (MSN/LB weight ratio of 1:2) at 60 °C and sonicated at 37 °C for 1 h. Then, the particles were centrifuged and washed with PBS three times to obtain the LB-MSN-GEM/HNK.

Syndecan-1 was conjugated to the LB-MSN-GEM/HNK by an amidation reaction. The above-prepared LB-MSN-GEM/HNK nanoparticles were dispersed in 1 mL PBS, mixed with NHS (1 mM, 50 μL) and EDC (1 mM, 50 μL), and reacted for 4 h at room temperature with stirring. Then, 50 μL of syndecan-1 (0.05 mM) was added to this suspension and mixed for 24 h at 4 °C. Next, the particles were centrifuged and washed with PBS three times. Finally, the washed particles were resuspended in 1 mL PBS to obtain the SDC1-LB-MSN-GEM/HNK.

The conjugation of SDC1 to the LB-MSN-GEM/HNK was confirmed by SDS-PAGE separation with Coomassie blue staining. The morphology and the mesoporous structure of the samples were observed using a transmission electron microscopy (TEM JEOL JEM-1200EX, Tokyo, Japan). N_2_ adsorption-desorption isotherms were measured on a Micromeritics ASAP2460 analyzer (Norcross, GE, USA). Sample sizes and zeta potentials were determined by a Nicomp 380 ZLS particle sizing system (Santa Barbara, CA, USA).

In vitro release of GEM and HNK from LB-MSN-GEM/HNK and SDC1-LB-MSN-GEM/HNK was evaluated by a dialysis method. First, 1.0 mL of LB-MSN-GEM/HNK or SDC1-LB-MSN-GEM/HNK was added to the dialysis bag (12,000–14,000 Da) and shaken in 30 mL of pH 7.4 PBS containing 0.2% Tween-80 at 37 °C for 48 h. At specified time intervals, 1.0 mL of release solution was taken out and replaced with 1.0 mL of fresh release fluid. Finally, the concentrations of GEM and HNK were analyzed by UPLC (Shimadzu, Kyoto, Japan) using an ultraviolet detector, 268 nm for GEM and 294 nm for HNK. The mobile phase consisted of 85% acetonitrile and 15% water.

### 3.3. Cell Culture and Animals

The BxPC-3 cell line was obtained from the Institute of Basic Medical Sciences, Chinese Academy of Medical Sciences (Beijing, China). The BxPC-3 cells were cultured in RPMI 1640 medium supplemented with FBS (10%) and penicillin-streptomycin (100 IU/mL and 100 μg/mL) in 5% CO_2_ at 37 °C. The BALB/c male nude mice (6–8 weeks) were purchased from Liaoning Changsheng Biotechnology Co. Ltd. (Benxi, China) and raised in a specific pathogen-free environment. 

### 3.4. Cellular Uptake

A quantitative determination of the cellular uptake was analyzed by flow cytometry. BxPC-3 cells were seeded in 6-well plates at a density of 5 × 10^5^ per well and cultured overnight. Then, the cells were incubated with free Cou6, LB-MSN-Cou6, and SDC1-LB-MSN-Cou6 in serum-free RPMI 1640 medium (final concentration of Cou6: 50 ng/ mL) for 2 h at 37 °C. Finally, the cells were rinsed with cold PBS, collected, and suspended in PBS. The mean fluorescence intensities of Cou6 were measured by a FACScan flow cytometer (BD FACSCalibur, Franklin Lakes, NJ, USA). In the competition experiments, 200 μg/mL SDC1 in serum-free RPMI 1640 medium was incubated with the BxPC-3 cells for 2 h before the SDC1-LB-MSN-Cou6 was added. 

For confocal microscopy analysis, BxPC-3 cells were seeded in glass-base dishes at a density of 3 × 10^5^ per well and cultured overnight. Then, the cells were incubated with free Cou6, LB-MSN-Cou6, and SDC1-LB-MSN-Cou6 in serum-free RPMI 1640 medium (final concentration of Cou6: 50 ng/mL) for 2 h at 37 °C. The competition experiment was the same as the flow cytometry assay. Finally, the cells were rinsed with cold PBS, fixed with 4% paraformaldehyde, and cell nuclei stained with Hoechst 33258. Finally, the fixed cells were observed using an LSM710 laser confocal microscope (Zeiss, Germany).

### 3.5. In Vitro Cytotoxicity Assays

Cytotoxic activities of LB-MSN-GEM/HNK, SDC1-LB-MSN-GEM/HNK, free GEM, free HNK, and GEM/HNK toward BxPC-3 cells were evaluated by SRB assay. BxPC-3 cells were seeded in 96-well plates at a density of 5 × 10^3^ cells/well and cultured overnight. Fresh medium containing varying concentrations of GEM/HNK (1.25/0.3125, 2.5/0.625, 5/1.25, 10/2.5, 20/5, 30/7.5, 40/10 ng/mL/μg/mL) formulations were added. After 48 h incubation, cells were fixed with 10% cold trichloroacetic acid and stained with 0.4% SRB to quantify the living cells. Then, 10 mM Tris base was added. Finally, the 96-well plates were placed in a microplate reader (Tecan Safire2, Mannedorf, Switzerland) to measure absorbance at 540 nm.

### 3.6. Apoptosis Assays

The effects of GEM/HNK on apoptosis were further verified by an annexin V-FITC and PI double-staining kit (Beyotime Biotechnology, Shanghai, China). Briefly, BxPC-3 cells were cultured with fresh medium containing various GEM/HNK (20 ng/mL/5 μg/mL) formulations for 24 h. After staining with Annexin V-FITC/PI, the cellular fluorescence was evaluated using flow cytometry to analyze cell apoptosis.

### 3.7. Measurement of Mitochondrial Membrane Potential (MMP), ATP, and Reactive Oxygen Species (ROS)

The MMP of BxPC-3 cells were measured according to the manufacturer’s protocol. The BxPC-3 cells were treated with LB-MSN-HNK, SDC1-LB-MSN-HNK, and free HNK (8 μg/mL) for 24 h. For flow cytometry measurement, the cells were collected and incubated with JC-1 working solution at 37 °C for 20 min, washed with buffer 2 times, and measured by flow cytometry. For confocal microscopy analysis, after being treated with LB-MSN-HNK, SDC1-LB-MSN-HNK, and free HNK, cells were washed with PBS, incubated with JC-1 working solution at 37 °C for 20 min, and washed 2 times with buffer, and the fluorescence imaging by laser confocal microscope was then taken immediately.

Similarly, BxPC-3 cells were seeded, cultured, and treated with drugs according to the MMP method. After treatment with drugs for 24 h, total protein was extracted and protein content was detected by the BCA assay. Then, 20 μL of protein was added to 100 μL ATP working solution, and the chemiluminescence RLU value of the solution was measured immediately with a microplate reader.

ROS was detected by fluorescence probe DCFH-DA. The BxPC-3 cells were treated with LB-MSN-HNK, SDC1-LB-MSN-HNK, and free HNK (8 μg/mL) for 24 h. Then, the cells were washed with PBS, incubated with 1 mL of DCFH-DA for 3 h at 37 °C, and washed 3 times with PBS, and the fluorescence imaging by LSM710 laser confocal microscope (Zeiss, Oberkochen, Germany) was taken immediately.

### 3.8. In Vivo Biodistribution Study

BxPC-3 cells were suspended in PBS and Matrigel (1:2) solution, and 5 × 10^6^ cells in 100 μL of cell suspension were administered subcutaneously into the left upper armpit of mice to establish xenograft tumor models. After the tumor volume approached approximately 200 mm^3^, mice were randomly assigned to two groups (*n* = 3). Tumor volume was calculated using the formula volume = 1/2 × length × width^2^. LB-MSN-DiR and SDC1-LB-MSN-DiR were injected into the mouse by the tail vein (5 μg DiR/mouse). Fluorescent images of the nude mice were scanned by IVIS Lumina Series III in vivo imaging system (Perkin Elmer, Waltham, USA) at 0 h, 2 h, 4 h, 6 h, 8 h, 12h, 24 h, 36 h, and 48 h. After 48 h, mice were sacrificed, and tumors and the major organs were excised for ex vivo fluorescence imaging.

### 3.9. In Vivo Antitumor Activity

Similarly, for in vivo antitumor activity assay, a xenograft tumor model was established; after the tumor volume approached approximately 150 mm^3^, mice were randomly assigned to six groups (*n* = 6). LB-MSN-GEM/HNK, SDC1-LB-MSN-GEM/HNK, free GEM/HNK, free GEM, free HNK, and physiological saline were injected into each mouse by the tail vein. The dosages of GEM and HNK were all 10 mg/kg, and the formulations were injected once every 3 days for a total of 4 times. The body weight and tumor size were measured after the day of injection. After 12 days of treatment, mice were sacrificed, and xenograft tumors and the major organs were excised. The major organs were fixed in 10% buffered formalin for H&E studies. Tumor tissues were weighed, photographed, and fixed in 10% buffered formalin. Then, the tumor tissues were performed for H&E, TUNEL, and IHC staining studies.

### 3.10. Statistical Analysis

All data were shown as mean ± standard deviation (SD). Student’s *t*-tests for two groups were analyzed for statistical comparisons. *p* < 0.05 and *p* < 0.01 represent significant differences of the two groups.

## 4. Conclusions

In summary, tumor- and stroma-targeted lipid bilayer-coated mesoporous silica nanoparticles were fabricated to co-deliver GEM and HNK for combating pancreatic cancer. SDC1 was first grafted on the LB-MSN to further improve the stroma and tumor targeting of nanoparticles through SDC1/IGF1R receptor-mediated endocytosis. In vitro studies demonstrated that the SDC1-LB-MSN-GEM/HNK exhibited higher cellular uptake, stronger cytotoxicity, and more obvious apoptosis-inducing effects than the tested LB-MSN-GEM/HNK. Mechanism studies revealed that the SDC1-LB-MSN-GEM/HNK induced apoptosis of BxPC-3 pancreatic cancer cells by regulating the mitochondrial apoptosis pathway, as evidenced by decreasing MMP and ATP and increasing the level of ROS. Compared with mice treated with the free drug and LB-MSN-GEM/HNK treatment, tumor growth was significantly inhibited with SDC1-LB-MSN-GEM/HNK treatment, which was induced by superior depletion of dense stroma and the synergistic GEM and HNK to effectively kill cancer cells. Overall, this study demonstrated that the combination of a first-line chemotherapy drug with a traditional Chinese medicine monomer with the help of a nanoparticle carrier in targeting depletion of the tumor stroma is a very promising strategy for pancreatic cancer treatment.

## Data Availability

The data presented in this study are available on request from the corresponding author.
